# Can we use a simple blood test to reduce unnecessary adverse effects from radiotherapy by timely identification of radiotherapy-resistant rectal cancers? MeD-Seq rectal study protocol

**DOI:** 10.1186/s12885-023-11671-y

**Published:** 2023-12-04

**Authors:** D. M. Mens, J. M. van Rees, S. M. Wilting, C. Verhoef

**Affiliations:** Erasmus MC University Medical Center, Rotterdam, Netherlands

**Keywords:** Rectal cancer, Chemoradiation, DNA-methylation

## Abstract

**Background:**

Chemoradiation therapy (CRT) followed by surgery is currently the standard of care to treat patients with locally advanced rectal cancer (LARC). CRT reduces local recurrences, but is associated with significant damage to the surrounding healthy tissue that can severely impact patients quality of life. Additionally, a proportion of patients (hardly) benefit from CRT. We aim to develop a diagnostic innovation, using DNA-methylation, which can enable a more selective and thereby more effective use of the available therapies for rectal cancer patients.

**Methods:**

MeD-Seq Rectal is a prospective single centre, observational study. 75 patients diagnosed with rectal cancer and will receive CRT as neoadjuvant treatment are will be included. DNA-methylation profiling will be performed on liquid biopsies to predict pathological response to CRT.

**Discussion:**

To data no clinical or image-based features were found that predict response to CRT. we hypothesize that DNA methylation patterns in liquid biopsies may provide a promising and patient-friendly strategy to predict CRT resistance upfront.

**Trial registration:**

This trial is registered at ClinicalTrials.gov (NCT06035471).

## Background

Current guidelines in the Netherlands recommend to treat patients with locally advanced rectal cancer (LARC) with pre-operative radiotherapy combined with radio-sensitizing low-dose chemotherapy (CRT), followed by surgery. CRT clearly reduces the local recurrence rate in patients with LARC also when combined with newer surgical techniques such as the total mesorectal excision (TME) [1, 2]. However, with these newer surgical techniques the local recurrence rate is already below 10% and CRT only results in an absolute reduction of about ~ 5%. Effects of CRT on the development of distant metastases and overall survival appear to be limited at best. On the other hand, CRT can induce significant damage to the surrounding healthy tissue which may complicate subsequent surgeries and gives rise to late adverse effects severely impacting quality of life in patients treated for rectal cancer [3]. Although these late adverse effects are not reported systematically by all randomised controlled trials, irradiated patients significantly more frequently suffer from bowel - (up to ~ 60% of patients), and sexual dysfunction (~ 30% of patients) more than 6 months after surgery [3]. In addition, CRT was shown to almost double the risk for secondary malignancies after more than 5 years of follow up [2, 4]. Based on data from 4 randomized controlled trials Bakx et al. estimate that 5–13% of patients experience benefit from CRT without any harm, and 0–2% of patients have both benefit and harm. The majority of patients (74–87%) experiences neither benefit nor harm of CRT, whereas 6–11% of patients are only harmed by CRT without any benefit [5]. Notwithstanding the added value of CRT in a fraction of rectal cancer patients, tools that allow for careful patient selection are needed to reduce the number of patients that currently receive CRT without benefit but with potential serious long-term adverse effects. We hypothesize that detection of DNA methylation patterns in liquid biopsies may provide a promising and patient-friendly strategy to predict CRT resistance upfront.

## Methods

### Objectives

Primary Objective: To investigate whether we can predict pathological response to preoperative CRT by DNA methylation profiling in liquid biopsies. This study is still exploratory and will not have clinical consequences on the included patients of this study. The short term aim for this study is to find DNA methylation patterns in liquid biopsies to predict pathological response to CRT. If DNA methylation patterns can be found in this study to predict pathological response to CRT, in the future patients can be selected to receive CRT and patients can be saved from potential side effects when they are found to be non-responders to CRT.

Secondary Objective(s): To determine whether blood sampling after 1 week of CRT provides additional relevant information with respect to pathological response, and to investigate the associations between cell-free DNA (cfDNA) methylation and molecular characteristics of the tumour tissue. To investigate molecular characteristics of patients with a clinical complete response with a watch and wait approach after CRT.

### Study design

Observational, single centre, prospective study.

### Study population

#### Population

Locally advanced rectal cancer patients scheduled for preoperative CRT.

### Inclusion criteria

In order to be eligible to participate in this study, a subject must meet all of the following criteria:

- Age ≥ 18 years.
- Histologically confirmed rectal adenocarcinoma.
- Scheduled for neoadjuvant chemoradiation.
- Written informed consent.

### Exclusion criteria

A potential subject who meets any of the following criteria will be excluded from participation in this study:

- Scheduled to another neoadjuvant schedule that comprises systemic chemotherapy or short-course radiation.
- Not able to read or understand Dutch language or mentally not capable.

### Sample size calculation

To detect a 2.5 fold difference in the chance of responding to CRT between circulating tumour DNA methylation high and - low patients with an alpha of 0.05 and a beta of 0.8, we need to include at least 64 patients according to the Chi-squared statistic with the continuity correction applied to compare proportions with a dichotomous outcome between two samples (http://www.sample-size.net/sample-size-proportions/). Based on previous and on-going studies we expect ~ 15% dropout due to either technical failure or loss to follow up. Therefore we will aim to include 75 patients in this project.

### Main study parameter/endpoint

Pathological response rate (percentage necrosis in comparison to tumour cells in the resected specimen) after CRT, determined by an expert pathologist according to the Mandard Tumour Regression Grade (TRG).

### Secondary study parameters/endpoints

cfDNA methylation profiling and molecular characteristics of the tumour tissue.

The blood sample obtained one week after initiation of the neo-adjuvant treatment will be utilized to investigate whether the dynamics in circulating tumour DNA (ctDNA) can be employed to early assess response to this therapy, and whether this provides additional value beyond conducting only a baseline measurement. The blood samples obtained prior to surgery and two weeks post-surgery may be utilized in the future to correlate ctDNA with the secondary endpoints of recurrence-free survival and overall survival. This aspect has been explicitly discussed with our patient advocates, who expressed no concerns. In a broader context, translational research on the potential applications of liquid biopsies benefits from longitudinal sample collection, enabling the examination of the effects of various treatment modalities on the quantity of ctDNA and its ultimate clinical relevance.

Clinical features and molecular characteristics of patients with a clinical complete reponse. The analyses of this tissue will be as described above for the other tissue but this group will be investigated separately.

### Other study parameters

Patient characteristics: age, sex, length, weight, cTNM, WHO performance status, extramural vascular invasion, mesorectal fascia involvement, lateral lymph node involvement.

### Recruitment and consent participants

Patients will be asked to participate in this study by their treating physician. If the patient is interested in participation, information concerning the research purpose will be explained. They also receive information in writing (informed consent form). Patients are allowed to sign the informed consent form immediately after careful reading, if a patient needs to consider their informed consent, this will be allowed as well until the start of CRT. Prior to any study related intervention (i.e. blood sample collection when blood sampling is already clinically indicated) the informed consent form must be signed by the patient and the person who has conducted the informed consent procedure.

### Randomisation, blinding and treatment allocation

Not applicable, as no randomisation or blinding is performed.

### Study procedures

Patients with locally advanced rectal cancer scheduled to receive pre-operative chemo-radiation therapy (CRT) in the Erasmus MC will be informed about the study by the local investigator or local study coordinator. From 75 consenting patients, blood (2 × 10 ml) will be collected in cell-stabilizing blood collection tubes at 4 time points: [1] After diagnosis and before start with CRT, [2] between 1 and 2 weeks after treatment, [3] 0–3 days before surgery, and [4] between 2 and 5 weeks after surgery at the outpatient clinic visit. Blood will be collected at time points 3 and 4 for translational research. Of note, most patients (including the involved patient representatives) do not consider additional blood draws as a burden. We will ensure blood draws for the current project will coincide with hospital visits as much as possible. Next to the blood sampling we will also retrieve the formalin-fixed paraffin-embedded (FFPE) tissue biopsy obtained at diagnosis from the pathology archive for participating patients to allow comparative analyses between blood and tumour tissue. Clinical (TNM stage, tumour location, age, gender), pathological (differentiation grade, MSI status, TRG), and follow up (RFS and OS) data will be collected for all participating patients from the electronic patient files. Information about pathology material will be stored in databases from IKNL, CBS and PALGA and can be used for further information in the future. Obtained molecular results will not be communicated with the participating patients on an individual basis as this may cause unnecessary anxiety, but overall study results will be shared.

Blood will be processed into plasma within 96 h and stored at -80 degrees, according to our standard pipeline. cfDNA will be isolated from of the stored plasma, quantified, and quality will be evaluated using standard laboratory techniques.

Similarly, we will isolate RNA and DNA from diagnostic FFPE-tissue sections and evaluate the quantity and quality.

MeD-seq analysis will be done on 10 ng cfDNA from the pre-treatment sample (baseline) and after 1 week of treatment as well as on genomic DNA from the tissue specimens as described before [6, 7]. In short, DNA will be digested with LpnPI yielding 32 bp fragments around the fully methylated recognition site containing a CpG. Samples will be prepped and purified for sequencing. Multiplexed libraries will be sequenced for ~ 20 M single reads. Tissue-based MeD-seq profiles will be used to determine the most informative regions for radio-sensitivity.

RNAseq analysis will be performed on RNA isolated from the FFPE tissue specimens, which has already been successfully performed in other, technically comparable, sample cohorts (unpublished data). RNAseq data will be used to determine genes associated with radio-sensitivity and, in addition, baseline molecular characteristics of the tumour, such as hypoxia, proliferation state of the tumour [8], and the consensus molecular subtype (CMS) [9] can be inferred from this data.

For RNAseq data, raw sequencing data will be mapped to the reference genome (GRCh38) using the STAR aligner after which the duplicate reads are marked using Sambamba. Next, FeatureCounts will be used to obtain read counts for each gene. These read counts will be normalized using GeTMM to obtain the gene expression levels that can be used for downstream analyses, such as hypoxia and proliferation scores [8], as well as consensus molecular sutype (CMS) calling [9].

For MeD-seq data, processing is carried out using specifically created scripts in Python, which include a trimming step to remove the Illumina adapters and a filtering step based on LpnPI restriction site occurrence between 13 and 17 bp from the 5’- or 3’ end of the read. Reads passing the filter are mapped to the genome using Bowtie 2. Using all unambiguously mapped reads count scores will be assigned to each individual LpnPI site in the genome. From genome-wide individual LpnPI site scores count scores are generated for 2 kilobase regions surrounding all known transcription start sites using a sliding window approach. To normalize potential differences in sequencing yields, regional read counts are divided by the total read count per sample. To generate a summary cfDNA methylation score, Z scores relative to healthy control samples (data already available from data of anonymous patients who donated blood at Sanquin and gave informed consent) will be calculated for every TSS region, which we will square and sum into a genome-wide Z score.

cfDNA methylation profiles and the summary score will be compared between patients with and without pathological response to CRT and, additionally, we will investigate associations between the cfDNA methylation profile/summary score, clinical, and molecular characteristics of the tumour.

### Statistical analysis

All main analyses will be performed according to the intention to treat principle using appropriate statistical software, such as R Project for Statistical Computing version 4.0.4. (https://www.r-project.org/) and SPSS version 21.0 (SPSS inc., Chicago, IL). ctDNA methylation levels will be scored high/low relative to the median value in the cohort. Missing samples will not be replaced, because a dropout rate taken into account in the sample size calculation.

We will perform adaptive group-regularized logistic ridge regression followed by post hoc group-weighted elastic net feature selection as described before [10]. In this innovative method group-regularisation of all markers is based on auxiliary data that is provided separately to the model resulting in improved marker selection. In our case this auxiliary data will consist of tissue-based comparison measures like fold changes in DNA methylation and RNA expression between patients with and without a pathological response. When sufficiently promising a targeted assay can be developed for the most optimal marker panel to reduce costs and facilitate clinical implementation.

Obtained data from patients following a watch and wait approach due to a sustained clinical response will be analysed as a separate subgroup since in these patients no pathological response can be determined. These patients will also follow the regular follow-up and will be analysed conform the other patients, only as a subgroup.

### Primary study parameter(s)

cfDNA methylation profiles and the summary score will be compared between patients with and without pathological response to CRT and, additionally, we will investigate associations between the cfDNA methylation profile/summary score, clinical, and molecular characteristics of the tumour. To obtain the most optimal panel of methylation markers predicting pathological response to CRT, we will perform adaptive group-regularized logistic ridge regression followed by post hoc group-weighted elastic net feature selection as described before [18]. In this innovative method group-regularisation of all markers is based on auxiliary data that is provided separately to the model resulting in improved marker selection. In our case this auxiliary data will consist of tissue-based comparison measures like fold changes in DNA methylation and RNA expression between patients with and without a pathological response. When sufficiently promising a targeted assay can be developed for the most optimal marker panel to reduce costs and facilitate future clinical implementation pending external validation of our findings. When available for the selected markers we will use publicly available data for this purpose and otherwise we will contact our network to find/collect a suitable sample cohort.

### Secondary study parameter(s)

In an exploratory analysis we will investigate whether the obtained methylation profiles are associated with our secondary outcome measures Recurrence Free Survival (RFS) and Overall Survival (OS). For this purpose we will perform univariate Cox regression analyses with the resulting methylation summary score for the entire profile as well as the selected marker panel (both continuous and dichotomized on the median). In case of significance (p < 0.1) for the univariate analysis subsequently a multivariate model will be built using known risk factors, including clinical (TNM stage, tumour location, age, gender), and pathological (differentiation grade, microsatellite instability status, TRG) parameters.

### Other study parameters

In an exploratory analysis we will investigate the methylation profile and treatment response with known clinical risk factors and patient characteristics. Multivariate regression analysis will be performed and also RFS and OS will be measured. Significance is determined to be (p < 0.05). Demographics and baseline characteristics will be summarized descriptively. Descriptive statistics will include median and interquartile range or mean and standard deviation for continuous variables, and frequency counts with percentages for categorical variables. Demographics and baseline characteristics will be summarized.

### Handling and storage of data and documents

After a patient has signed the informed consent form, the patient will be registered by the study coordinator. The study coordinator will give each patient a unique subject number (i.e. not related to patients initials, date of birth or other identifiers), starting from MeD-001. The key to the patient subject number is safeguarded by the local investigator. The registration form will at least include the patients’ subject number, the name of the treating physician and investigator, a statement the patient meets all selection criteria and the date of written informed consent. All patient samples will be coded with the patients’ subject number and unique lab code. The original signed informed consent form will be kept on site in the Investigators Site File. The registration form and the collected patient data will be recorded in a digital database.

Research data will be stored in a Castor database and will be handled confidentially. Research data that can be traced to individual persons can only be viewed by authorized personnel. These persons are the members of the research team, members of the health care inspection, and members of the Medical Research Ethics Committee of the participating hospital. Data review may be necessary in order to ensure the reliability and quality of the research. The handling of personal data is in compliance with the Dutch Data Protection Act (in Dutch: Uitvoeringswet ‘Algemene Verordening Gegevensbescherming).

Collected data, essential documents, source documents and human materials (i.e. blood samples) will be stored for at least 15 years in such a manner that they are protected from accidental loss and can easily be retrieved for review. Essential documents are those documents that permit evaluation of the conduct of a trial and the quality of the data produced. The essential documents may be subject to, and should be available for, audit by the Sponsor’s auditor and inspection by the regulatory authority(ies).

## Discussion

To data no clinical or image-based features were found that predict response to CRT. Tissue-based analyses have been performed to find predictive molecular biomarkers for response to CRT, but to date none of the identified potential markers has been clinically implemented [11]. Gene expression panels were identified that allow for the discrimination between responders and non-responders [12, 13]. However, the need for fresh frozen pre-treatment tissue biopsies with sufficient tumour material hampers validation and subsequent clinical implementation of this type of predictive marker. Next to distinct gene expression profiles, high global DNA methylation levels in pre-treatment biopsies were associated with a poor response to CRT [14]. A biological rationale for this observation was provided by Thienpont et al., who showed that tumour-specific DNA methylation increases under hypoxia, which represents one of the major driving mechanisms behind radiotherapy resistance [8]. In contrast to RNA expression, DNA methylation provides a stable and robust biomarker, which facilitates subsequent validation and clinical implementation. Blood is known to contain circulating cfDNA from dying cells in the body, including ctDNA, rendering so-called liquid biopsies (i.e. blood sampling) a patient-friendly alternative to the traditional tissue biopsy. In addition, liquid biopsies are believed to more accurately reflect potential intra-tumour heterogeneity compared to a single tissue biopsy. The challenge is to distinguish ctDNA fragments from the total pool of cfDNA present in the blood. To date this is predominantly done using tumour-specific DNA mutations, limiting its application to patients carrying a known mutation in the tumour. Notwithstanding this limitation recent studies have shown that detection of ctDNA in the blood is predictive for disease recurrence [15], suggesting liquid biopsies could play a key role in the clinical management of rectal cancer patients. Data on the use of liquid biopsies to determine a tumour’s radio-sensitivity upfront is limited [16], but preliminary work suggests that high ctDNA levels before treatment start are associated with subsequent unresponsiveness of rectal cancer to chemo-radiation therapy. In addition, a spike in the ctDNA level directly after treatment start could reflect response to cytotoxic therapies such as radiotherapy [17]. Recent work by others and us demonstrates that tumour-derived DNA methylation profiles are also detectable in a patient’s blood [7], which is of particular interest for the current proposal considering the above mentioned link between increased DNA methylation levels and radiotherapy resistance. An additional advantage of DNA methylation over currently widely used mutations is the high prevalence of altered DNA methylation markers compared to mutations. Symonds et al. showed that ctDNA could be detected in 70% of pre-treatment blood samples from rectal cancer patients using only 2 methylation markers [18]. In conclusion, we hypothesize that DNA methylation patterns in liquid biopsies may provide a promising and patient-friendly strategy to predict CRT resistance upfront.

## Data Availability

Research data will be stored in a Castor database and will be handled confidentially. Research data that can be traced to individual persons can only be viewed by authorized personnel. Information about pathology material will be stored in databases from IKNL, CBS and PALGA and can be used for further information in the future.

## Abbreviations

CBS: Central Statistical Office
in Dutch: Centraal Bureau voor Statistiek
cfDNA: Cell-free DNA
CRT: Chemoradiation therapy
ctDNA: Circulating tumour DNA
IKNL: Netherlands Comprehensive Cancer Organisation; in Dutch: Integraal Kankercentrum Nederland
FFPE: Formalin-fixed paraffin-embedded
LARC: Locally advanced rectal cancer
METC: Medical research ethics committee (MREC); in Dutch: Medisch-Ethische Toetsings Commissie (METC)
OS: Overall survival
PALGA: Pathological Anatomy National Automated Archive; in Dutch: Pathologisch Anatomisch Landelijk Geautomatiseerd Archief
RFS: Recurrence Free Survival
TME: Total Mesorectal Excision
TNM: The TNM Classification of Malignant Tumors
TRG: Tumour Regression Grade
